# Combination of Irreversible Electroporation and *Clostridium novyi*-NT Bacterial Therapy for Colorectal Liver Metastasis

**DOI:** 10.3390/cancers17152477

**Published:** 2025-07-26

**Authors:** Zigeng Zhang, Guangbo Yu, Qiaoming Hou, Farideh Amirrad, Sha Webster, Surya M. Nauli, Jianhua Yu, Vahid Yaghmai, Aydin Eresen, Zhuoli Zhang

**Affiliations:** 1 Department of Radiological Sciences, University of California, Irvine, CA 92868, USA; 2 Department of Biomedical Engineering, University of California, Irvine, CA 92697, USA; guangboy@uci.edu; 3 Department of Biomedical and Pharmaceutical Sciences, Harry and Diane Rinker Health Science Campus, Chapman University, Irvine, CA 92618, USAshwebster@chapman.edu (S.W.);; 4 Department of Medicine, University of California, Irvine, CA 92868, USA; 5 Chao Family Comprehensive Cancer Center, University of California, Irvine, CA 92868, USA; 6 Department of Pathology & Laboratory Medicine, University of California, Irvine, CA 92617, USA

**Keywords:** artificial intelligence, *Clostridium novyi*-NT, colorectal cancer liver metastasis, irreversible electroporation, magnetic resonance imaging

## Abstract

Colorectal liver metastasis (CRLM) poses a significant challenge in oncology due to its high incidence and poor prognosis in unresectable cases. Current treatments, including surgical resection, systemic chemotherapy, and liver-directed therapies, often fail to effectively target hypoxic tumor regions, which are inherently more resistant to these interventions. This review examines the potential of a novel therapeutic strategy combining irreversible electroporation (IRE) ablation and *Clostridium novyi*-nontoxic (*C*. *novyi*-NT) bacterial therapy. IRE is a non-thermal tumor ablation technique that uses high-voltage electric pulses to create permanent nanopores in cell membranes, leading to cell death while preserving surrounding structures, and is often associated with temporary tumor hypoxia due to disrupted perfusion. *C*. *novyi*-NT is an attenuated, anaerobic bacterium engineered to selectively germinate and proliferate in hypoxic tumor regions, resulting in localized tumor cell lysis while sparing healthy, oxygenated tissue. The synergy between IRE-induced hypoxia and hypoxia-sensitive *C*. *novyi*-NT may enhance tumor destruction and stimulate systemic antitumor immunity. Furthermore, the integration of advanced imaging and artificial intelligence can support precise treatment planning and real-time monitoring. This integrated approach holds promise for improving outcomes in patients with CRLM, though further preclinical and clinical validation is needed.

## Simple Summary

Colorectal liver metastasis (CRLM) presents a significant challenge, especially in patients unsuitable for surgery, due to the limited success of the current treatments for targeting the hypoxic tumor regions effectively. This review highlights a promising new strategy combining irreversible electroporation (IRE) and *Clostridium novyi*-NT (*C*. *novyi*-NT) bacterial therapy. IRE ablates tumors and temporarily induces hypoxia, creating an ideal environment for *C*. *novyi*-NT, an engineered bacterium that specifically destroys cancer cells in low-oxygen conditions. This synergistic approach aims to enhance tumor destruction and stimulate the immune response. While needing further validation, integrating advanced imaging and artificial intelligence for precise planning, this novel treatment offers a potential breakthrough for CRLM patients.

## 1. Introduction

Colorectal cancer is a leading global malignancy, with an estimated 1.9 million new cases and 935,000 deaths [1]. The liver is the primary site of metastasis, with 25–50% of colorectal cancer patients developing colorectal liver metastasis (CRLM), significantly worsening prognosis [2]. CRLM is a major cause of colorectal cancer-associated mortality, with five-year survival rates of 30–58% for resectable and less than 10% for unresectable cases [3]. Surgical resection is the only curative treatment; however, only 10–20% of patients are eligible at diagnosis due to the size and location of the tumors, the number of lesions, or extrahepatic disease [4]. For unresectable CRLM, systemic chemotherapy, often combined with targeted therapies, e.g., bevacizumab or cetuximab, extends median overall survival to 20–24 months; however, its efficacy is restricted by resistance and toxicity [4].

Hypoxia, characterized by a low oxygen level in the tumor microenvironment (TME), is a hallmark of CRLM and a major barrier challenging the therapeutic efficacy [5]. Hypoxic regions resist chemotherapy and radiation due to poor vascularization and altered metabolism, promoting resistance through mechanisms including upregulation of hypoxia-inducible factors [6]. This has driven the development of novel therapies targeting hypoxic areas and stimulating systemic anti-tumor immunity.

Two emerging approaches, irreversible electroporation (IRE) ablation and *Clostridium novyi*-nontoxic (*C*. *novyi*-NT) bacterial therapy, show promise as a novel combinatorial therapeutic strategy against CRLM [7,8]. IRE ablation uses high-voltage electrical pulses to disrupt cell membranes, while preserving critical structures and inducing immune responses [9]. IRE ablation can induce temporary hypoxia, primarily through transient vascular occlusion and disruption of capillary blood flow. Thus, the resulting environment can be specifically targeted by *C*. *novyi*-NT, an attenuated anaerobic bacterium that releases toxins to lyse cancer cells and trigger immunity [10]. Combining these therapies could enhance tumor destruction by leveraging hypoxia induced by IRE ablation to further promote *C*. *novyi*-NT colonization and activity within the TME. Moreover, artificial intelligence-powered dynamic monitoring of underlying characteristic changes during treatment can enable therapeutic regimen adjustments to further enhance treatment efficacy and ultimately facilitate enormous potential for personalized therapy.

This review examines current CRLM treatments, explores the potential synergy of IRE ablation combined with *C*. *novyi*-NT bacterial therapy (including their rationale), and discusses the role of imaging and AI-driven analysis for therapeutic monitoring to optimize outcomes. Moreover, it provides in-depth analyses of techniques, including portal vein embolization and associating liver partition and portal vein ligation for staged hepatectomy to expand resectability, addressing the specific mechanisms by which they induce future liver remnant hypertrophy.

## 2. Current Treatments for CRLM

### 2.1. Surgical Resection

Surgical resection is the primary curative treatment for CRLM, achieving five-year survival rates as high as 67% in carefully selected patients [11]. However, its applicability is limited by several factors, for example, multiple liver lesions, proximity to critical structures, or extrahepatic disease, with only 10–20% of patients qualified for the treatment based on the diagnostic metrics [4]. To increase the number of resectable cases, techniques such as portal vein embolization and associating liver partition and portal vein ligation are employed to optimize the future liver remnant hypertrophy, reducing the risk of post-hepatectomy liver failure [12]. Preoperative analysis of noninvasive medical imaging data (computed tomography (CT) and magnetic resonance imaging (MRI)) and functional liver assessments is crucial for surgical planning to ensure adequate future liver remnant volume and function [13]. However, even with meticulous planning and successful resection, recurrence remains a major challenge in the management of CRLM. Table 1 summarizes the advantages and limitations of current treatment strategies for CRLM.

### 2.2. Systemic Chemotherapy

Systemic chemotherapy remains the cornerstone of treatment for unresectable CRLM, offering a median overall survival of 20–24 months [14]. Standard first-line regimens typically involve combinations such as FOLFOX (folinic acid, fluorouracil, and oxaliplatin), FOLFIRI (folinic acid, fluorouracil, and irinotecan), and XELOX (capecitabine and oxaliplatin), frequently administered alongside targeted agents like bevacizumab (targeting vascular endothelial growth factor) or cetuximab (targeting epidermal growth factor receptor, EGFR) in RAS wild-type tumors [15,16]. RAS family genes play a pivotal role in promoting tumor cell growth, angiogenesis, and tumor invasiveness through the mitogen-activated protein kinase signaling pathway. The presence of RAS wild-type status in CRLM, where RAS mutations occur in approximately 40–50% of cases, is an important factor in determining patient eligibility for anti-EGFR therapies [17]. Furthermore, neoadjuvant chemotherapy plays a crucial role in downstaging initially unresectable tumors, facilitating subsequent surgical resection in a significant proportion of patients, ranging from 12% to 38% [18]. However, the efficacy of systemic chemotherapy is often hampered by the presence of hypoxic tumor regions, which exhibit inherent treatment resistance due to factors such as poor drug penetration resulting from compromised vasculature and the activation of hypoxia-inducible factor-mediated survival pathways [19]. This hypoxic microenvironment not only limits the cytotoxic effects of chemotherapy but can also promote angiogenesis, metastasis, and the selection of more aggressive, stem-like tumor cell populations. Moreover, the systemic toxicity associated with prolonged chemotherapy regimens often limits their long-term application and can significantly impact patients’ quality of life, frequently leading to dose reductions or treatment discontinuation [20]. To overcome these challenges, different strategies are under investigation, including the development of hypoxia-activated prodrugs that selectively release cytotoxic agents in oxygen-deficient environments, and the use of hypoxia-modifying agents aimed at re-oxygenating tumors or inhibiting hypoxia-inducible factor signaling pathways [21,22].

### 2.3. Liver-Directed Therapies

Liver-directed therapies provide important alternatives for patients with unresectable CRLM, particularly when systemic options are limited or in cases with chemotherapy-refractory conditions. These modalities aim to achieve local tumor control, prolong survival, and downstage tumors to resectability in some cases [23,24,25].

#### Transarterial Therapies

Transarterial chemoembolization (TACE) and selective internal radiation therapy (SIRT) exploit the liver’s unique dual blood supply to deliver high concentrations of chemotherapy or radiation directly to tumors while sparing normal liver tissue [26]. SIRT, which uses yttrium-90 (Y-90)-labeled microspheres, has demonstrated improved progression-free survival when combined with systemic chemotherapy, though randomized trials have not shown a significant overall survival benefit [27]. Median overall survival after SIRT for liver-dominant metastatic colorectal cancer is reported at approximately 15 months, with the best outcomes seen when used earlier in the treatment course [28].

Hepatic arterial infusion chemotherapy (HAIC) has been extensively investigated for the management of CRLM, particularly in inoperable patients, due to potential improvement of both local tumor response and overall survival (OS) when administered in conjunction with systemic chemotherapy. Early investigations demonstrated the efficacy of HAIC using floxuridine and dexamethasone combined with systemic irinotecan, reporting an objective response rate (ORR) of 74% and a median OS of 20 months [29]. A subsequent study further explored HAIC with floxuridine/dexamethasone alongside systemic oxaliplatin and either irinotecan or fluorouracil/leucovorin, achieving remarkable ORRs of 90% and 87% with median OS values of 36 months and 22 months, respectively [30]. Notably, 19% of patients in the irinotecan cohort achieved sufficient tumor regression to permit surgical resection. Building on these findings, subsequent investigations focused on HAIC as a conversion strategy in a neoadjuvant setting. A Phase I study assessing conversion to resectability involving 49 patients with unresectable CRLM treated with HAIC combined with systemic oxaliplatin and irinotecan, yielded a 92% ORR and a 47% overall CTR, with a higher resectability rate of 57% in chemotherapy-naïve individuals [31]. These findings were further validated by a prospective Phase II trial examining long-term outcomes in 64 patients receiving HAIC plus systemic therapy [32]. After a median follow-up of 81 months, the resection rate reached 52%, effectively doubling historical expectations, with a median progression-free survival (PFS) of 13 months and OS of 38 months; resected patients exhibited a significantly prolonged 5-year OS of 63% compared to 13% in non-resected patients. Similarly, a prospective investigation by Goéré et al. evaluated patients with unresectable CRLM treated with HAIC-oxaliplatin plus systemic 5-fluorouracil and leucovorin, achieving a 26% CTR in a cohort where 79% were previously treated [33]. Liver resection in this group resulted in a median OS of 41.7 months and a 5-year OS of 56%, with first-line HAIC leading to a significantly higher CTR of 53% compared to 19% in those receiving HAIC after systemic therapy failure. The European multicenter Phase II OPTILIV trial further supported the efficacy of HAIC-based triplet regimens, with 66 patients with high intrahepatic disease burden receiving HAIC with irinotecan, oxaliplatin, and 5-fluorouracil in combination with intravenous cetuximab [34]. This trial yielded an overall CTR of 30% and a median OS of 25.7 months, with stratified analysis demonstrating a 63% ORR in second-line patients, though reduced to 38% with a median OS of 15.2 months in third- or fourth-line patients [35].

TACE, while a well-established first-line therapeutic option for intermediate-stage HCC, has a more limited role in CRLM, typically reserved for liver-dominant, chemotherapy-refractory metastatic disease as a second-line or later therapy [36]. Conventional TACE (cTACE), adapted from HCC protocols, involves the temporary occlusion of tumor-feeding arteries and local drug delivery via an emulsion; however, its utility has diminished with the advent of drug-eluting bead technology (DEB-TACE) due to cTACE’s limited impact on OS and lack of prospective trial support. DEB-TACE, utilizing polymer microspheres preloaded with chemotherapeutic agents, enables controlled, sustained drug release, resulting in lower systemic drug exposure and reduced toxicity to healthy liver tissue compared to cTACE [37,38]. DEBIRI-TACE has emerged as a more standardized and promising approach for patients with chemo-refractory CRLM. In a prospective multicenter study of 55 patients undergoing a median of two DEBIRI sessions, an ORR of 75% was achieved, with median PFS and OS of 11 and 19 months, respectively [39]. In a separate study, Aliberti et al. analyzed outcomes in patients who had failed at least two prior systemic regimens, reporting an ORR of 78% at 3 months, with a median PFS of 8 months and OS of 25 months at a median follow-up of 29 months [40]. More modest results were reported by Izzei et al., who conducted a prospective Phase II study of 20 heavily pretreated patients, recording an ORR of 60%, with a median PFS of 4 months and 7.3 months of OS, possibly attributable to small sample size and high dropout rate [41]. Combination strategies with biological agents have also been evaluated; a prospective randomized study of 30 patients demonstrated that the addition of bevacizumab to DEBIRI significantly improved outcomes relative to DEBIRI alone, with increased ORR, PFS, and median OS [42].

Stereotactic body radiation therapy (SBRT) has emerged as a non-invasive local treatment modality delivering ablative, high-dose radiation with submillimeter precision to well-defined hepatic lesions, yielding 1-year local control rates exceeding 90% in carefully selected patients with limited hepatic metastatic disease [43]. This approach is particularly beneficial for surgically inoperable patients due to lesion location, comorbidities, or anatomical constraints; however, its long-term survival benefit and comparative efficacy against other local liver-directed therapies warrant further investigation [44]. Historically, the broader application of external beam radiation for hepatic tumors was limited by the low radiation tolerance of normal liver parenchyma, necessitating technological advancements for safe dose escalation [45].

SIRT has evolved as a viable alternative for unresectable liver malignancies, involving the intra-arterial infusion of radioactive microspheres that selectively target tumor vasculature while sparing healthy tissue [46]. Y-90, a pure beta-emitter with a mean tissue penetration of 2.5 mm and a short half-life of 64.2 h, is the most commonly used isotope due to its capacity to deliver high radiation doses with limited collateral toxicity [47]. While traditionally reserved for chemo-refractory liver-only or liver-dominant CRLM, the application of Y-90 SIRT has expanded to include first-line and neoadjuvant settings, with ORR ranging from 10% to 48% and median OS between 9.6 and 14.9 months [48,49]. A large retrospective series by Saxena et al. involving 302 patients undergoing resin Y-90 SIRT for unresectable, heavily pretreated CRLM reported a median OS of 10.5 months and 24-month survival of 21%; multivariate analysis identified poor radiologic response, high hepatic tumor burden, extensive prior chemotherapy, and low baseline hemoglobin as adverse prognostic indicators [50]. A multicenter analysis by Kennedy et al. of 208 patients with prior chemotherapy failure demonstrated a 36% response on CT and a markedly higher 91% on PET imaging, highlighting the limitations of RECIST-based assessment for metabolic tumor response to Y-90 SIRT and reinforcing the value of PET-CT [51,52]. Collectively, the evidence supports Y-90 SIRT as a safe and effective liver-directed therapy with potential for integration into various lines of treatment for unresectable CRLM, especially when paired with accurate metabolic imaging and multidisciplinary management.

## 3. Alternative Therapies for CRLM

### 3.1. Ablation

Partial hepatectomy remains the gold-standard curative intervention for resectable solid liver malignancies [53]. However, high recurrence rates post-resection have spurred interest in alternative approaches such as ablation therapies. While thermal ablation was historically reserved for non-surgical candidates, current NCCN guidelines now endorse its use as a standalone or adjunctive therapy for select patients with small, margin-achievable tumors [54].

Partial hepatectomy remains the gold-standard curative intervention for patients with resectable solid liver malignancies, including CRLM [53]. However, the high rate of post-resection recurrence has spurred interest in minimally invasive, liver-preserving strategies such as image-guided ablation. Historically, thermal ablation was reserved for non-surgical candidates; however, current NCCN guidelines endorse its use as either a standalone or adjunctive therapy in patients with small, margin-negative tumors who are not ideal surgical candidates [54].

Ablation techniques, which include both thermal and non-thermal modalities, function by inducing targeted tumor cell death through energy delivery while also promoting localized immune activation. Notably, not all ablation modalities appear to elicit equivalent systemic immune responses. Emerging evidence suggests distinct immunomodulatory profiles between thermal ablation and non-thermal techniques such as IRE. Despite ongoing technological advancements, significant knowledge gaps persist regarding the comparative efficacy of ablation versus resection, particularly in patients with small-volume CRLM. The randomized LAVA trial, a multicenter study conducted in the UK and the Netherlands, was terminated early due to recruitment difficulties stemming from a lack of clinical equipoise and surgeon preference for resection. This underscores the importance of designing pragmatic trials that account for real-world practice patterns [55].

Radiofrequency ablation (RFA) and microwave ablation (MWA) are the most used thermal ablation techniques for CRLM. Both are indicated for tumors typically less than 3 cm in diameter and situated away from major vessels or critical structures [56,57,58]. MWA offers several technical advantages over RFA, including higher intratumoral temperatures, faster ablation times, and reduced susceptibility to the heat-sink effect, which occurs when heat is dissipated by blood flow in adjacent vasculature. This makes MWA more effective in treating larger tumors or those located near vessels. Meta-analyses demonstrate that MWA is associated with lower local tumor progression (LTP) rates and improved disease-free survival when compared to RFA, though both techniques remain safe and effective [57]. Nonetheless, recurrence rates rise significantly for tumors larger than 3 cm or in proximity to major vessels, primarily due to incomplete ablation and limitations in achieving uniform thermal coverage [59,60].

#### IRE Ablation

IRE ablation is a non-thermal technique that delivers high-voltage electrical pulses, creating nanopores in cell membranes and inducing apoptosis and necrosis via intracellular calcium release, mitochondrial permeability changes, and oxidative stress (Table 2) [61,62]. Unlike thermal ablation, IRE preserves critical extracellular matrix structures, including collagen scaffolds and vasculature [62,63]. This structural preservation facilitates rapid antigen release from ablated cells into intact vasculature, enhancing immune activation by exposing tumor-associated antigens and damage-associated molecular patterns that stimulate dendritic cells and cytotoxic T lymphocytes [64,65]. While IRE ablation reduces thermal damage risks, its effectiveness is limited to smaller tumors (≤30 mm diameter) and requires precise probe placement [9]. Furthermore, the potential for inducing arrhythmia necessitates general anesthesia, which can negatively impact treatment affordability.

The randomized phase II CLOCC trial demonstrated the clinical benefit of adding local therapy to systemic chemotherapy in patients with oligometastatic CRLM. Patients receiving RFA or resection in addition to systemic therapy had a median progression-free survival (PFS) of 16.8 months, compared to 9.9 months for those receiving systemic therapy alone [66]. Although CLOCC did not evaluate IRE specifically, the study supports the principle of local control in metastatic CRLM. However, extrapolating these findings to IRE is limited by selection bias, as IRE is generally reserved for anatomically challenging lesions or for patients who are not candidates for thermal modalities. Most IRE studies are small, heterogeneous, and non-randomized [62].

In one study by Hosein et al., IRE achieved a complete response in 36% and a partial response in 21% of treated CRLM, with a median follow-up of 11 months [67]. Repeat IRE ablation was associated with local control rates of 74–96% [62]. Across multiple retrospective cohorts, 1-year LTP rates following IRE ranged from 13.4% to 37%, comparable to the 1-year LTP rates reported for RFA (3–30%) and MWA (3–13%) [68]. IRE appears particularly effective for smaller lesions. For tumors < 3 cm, Cannon et al. reported a 1-year local recurrence-free survival (LRFS) of 98% [69], while Freeman et al. found a 100% LRFS for lesions < 2 cm [70]. However, Hosein et al. observed no size-dependent differences in outcomes [67], though the general consensus suggests a 5 cm upper limit for optimal results cm [69]. Elevated body mass index (BMI) may reduce IRE efficacy, likely due to altered tissue conductivity and difficulty in accurately delineating lesions in fatty livers [68].

Comparative analyses between IRE and other modalities, including surgery and SBRT, are confounded by selection bias, as IRE is often used in heavily pretreated patients or those with unresectable tumors [62]. CRLM are known to be relatively radioresistant, particularly following prior chemotherapy. A multicenter SBRT registry demonstrated the following declining local control rates over time: 87% in 1 year, 75% in 2 years, and 68% in 3 years. Outcomes in CRLM were notably poorer than in other histologies [68]. In contrast, the Amsterdam CORE registry reported superior median OS, local progression-free survival (LPFS), and local control for thermal ablation compared to SBRT (OS: 54.0 vs. 27.4 months) [70]. For tumors ineligible for thermal ablation, IRE is increasingly used as either a primary or salvage option, including following SBRT failure. SBRT remains a consideration only for patients with poor performance status [70].

Beyond cytoreduction, IRE may play a role in reshaping the tumor microenvironment. It transiently induces hypoxia within the ablation zone, suggesting opportunities for rational combination with hypoxia-targeted therapies. For instance, IRE-induced hypoxia could enhance the activity of hypoxia-activated prodrugs or facilitate colonization of anaerobic bacteria such as *Clostridium novyi*-NT, which preferentially proliferate in low-oxygen environments and exert cytotoxic effects. Moreover, the immunogenic cell death associated with IRE, characterized by DAMP release, could be further amplified under hypoxic conditions to boost systemic antitumor immune responses when used in conjunction with immunotherapy [71]. However, to date, no studies have systematically investigated this specific aspect as a means to enhance therapeutic efficacy in the treatment of CRLM. The absence of such data underscores a critical gap in the current literature and highlights the need for mechanistic and translational research to optimize treatment strategies and improve clinical outcomes in this patient population.

### 3.2. Bacterial Therapy

In recent years, bacterial-based cancer therapy has gained considerable attention as a novel and multifaceted approach for targeting solid tumors. Historically, certain bacteria have been linked to cancer development due to their capacity to induce chronic inflammation, disrupt host cellular processes, or produce carcinogenic metabolites. However, accumulating evidence has revealed that specific bacterial species, either naturally occurring or genetically engineered, can be leveraged to combat cancer through a variety of mechanisms. These include immune system stimulation, selective colonization of tumors, delivery of therapeutic agents, disruption of tumor metabolism, and direct tumor cell lysis [72]. Unlike conventional therapies that often struggle to penetrate hypoxic tumor cores or differentiate between malignant and healthy cells, bacterial therapies exploit the unique microenvironment of tumors, particularly the presence of necrotic and oxygen-deprived regions, to achieve targeted effects.

Several bacterial species have shown potential as therapeutic agents. Escherichia coli, for instance, has been observed to elicit CD8^+^ cytotoxic T cell responses that upregulate MHC class I on tumor cells and enhance immune-mediated tumor clearance [72]. Salmonella typhimurium and Listeria monocytogenes have been engineered to deliver tumor-associated antigens or cytotoxic payloads directly into tumors, triggering both innate and adaptive immune responses [72]. Likewise, Bifidobacterium longum and Lactobacillus species have been investigated for their ability to colonize tumors and modulate the host immune environment [72]. These approaches often rely on the bacteria’s ability to act as delivery vectors, enhancing antigenicity, recruiting immune effector cells, and inducing systemic immunity against tumor cells.

Despite the promise of these species, *C*. *novyi*-NT has emerged as a particularly compelling candidate due to its highly selective growth within hypoxic tumor regions and its potent immune-stimulating properties. As a strict anaerobe, *C*. *novyi*-NT spores germinate exclusively within the oxygen-deprived zones that are characteristic of solid tumors but absent in healthy tissue. This property provides a built-in safety mechanism that limits bacterial proliferation to the TME, reducing the risk of systemic toxicity [73]. Upon germination, *C*. *novyi*-NT not only lyses tumor cells directly but also releases pathogen-associated molecular patterns that trigger toll-like receptor signaling, leading to the production of pro-inflammatory cytokines such as IL-12, TNF-α, and IFN-γ. These cytokines, in turn, stimulate dendritic cell maturation, enhance CD8^+^ cytotoxic T lymphocyte responses, and activate natural killer cells, all of which are crucial for effective tumor immunosurveillance and destruction [73].

Compared to other Clostridium species, *C*. *novyi*-NT offers several advantages. Clostridium perfringens, for example, produces enterotoxins that can disrupt tight junctions in epithelial cells by targeting claudin proteins, while Clostridium botulinum secretes neurotoxins that modulate vascular tone in the TME. Although these effects can contribute to tumor suppression, they also carry significant risks due to systemic toxicity and off-target effects [74]. In contrast, *C*. *novyi*-NT has been genetically engineered to remove its lethal toxin, significantly improving its safety profile [75]. Moreover, its spores are efficiently cleared from systemic circulation via the reticuloendothelial system, minimizing the risk of persistent infection [75]. This refined safety, combined with the ability to induce localized tumor lysis and robust immune activation, makes *C*. *novyi*-NT particularly well-suited for use in immunotherapeutic strategies, especially when combined with immune checkpoint blockade, chemotherapy, or dendritic cell vaccination [76].

*C*. *novyi*-NT represents a next-generation bacterial immunotherapy agent with exceptional tumor selectivity, immune-stimulatory potential, and safety profile. Its ability to germinate in the necrotic cores of tumors, induce antitumor Th1 responses, and synergize with existing therapies distinguishes it from other bacterial platforms [76]. These features collectively support its selection as the lead candidate for bacterial-mediated cancer treatment in the context of solid tumors, particularly those that are immunologically “cold” or resistant to conventional interventions.

#### *C*. *novyi*-NT

*C*. *novyi*-NT, an attenuated anaerobic bacterium, exploits the hypoxic microenvironment prevalent in solid tumors, including CLRM, to germinate and exert oncolytic effects. Upon spore germination within these oxygen-deficient regions, *C*. *novyi*-NT secretes phospholipases and lipases that disrupt tumor cell membranes, induce cytotoxicity [10,73]. Simultaneously, it triggers a systemic anti-tumor immune response by releasing pro-inflammatory cytokines such as interleukin-6 and granulocyte-colony stimulating factor, alongside damage-associated molecular patterns, which activate dendritic cells and cytotoxic T lymphocytes [10,76]. This dual mechanism of action allows for precise targeting of hypoxic tumor niches while preserving normoxic tissues (Table 3).

Preclinical investigations in colorectal cancer models have demonstrated that *C*. *novyi*-NT, when combined with microtubule stabilizers or liposomal doxorubicin, can achieve rapid tumor regression, with complete eradication observed in a subset of xenografts [10]. Early-phase clinical trials in humans have shown promising signals, with partial responses reported in 22–25% of patients across a variety of advanced solid tumors, including nasopharyngeal and tongue squamous cell carcinomas [77]. Notably, studies in spontaneous canine sarcomas have yielded a 37.5% objective response rate, including three complete responses, highlighting the translational potential of this therapeutic approach [73]. Furthermore, murine studies have indicated that treatment with *C. novyi*-NT can lead to long-term cures in 25% to 30% of treated animals, an effect attributed to the development of an adaptive immune response that protects against subsequent tumor rechallenge [76].

Despite these promising findings, key hurdles remain in the clinical application of *C*. *novyi*-NT. Optimizing intra-tumoral delivery to ensure adequate spore penetration throughout the tumor mass is critical, as is effectively managing potential infection-related toxicities such as fever and tissue necrosis [77]. The inherent efficacy of *C*. *novyi*-NT is also critically dependent on the pre-existence of tumor hypoxia and necrosis to support robust bacterial germination and proliferation. Ongoing research efforts are focusing on combinatorial strategies to enhance its efficacy, such as pairing *C*. *novyi*-NT with immune checkpoint inhibitors like pembrolizumab, which have demonstrated synergistic anti-tumor activity in early-stage clinical evaluations [78]. Moreover, genetic engineering approaches aimed at enhancing the potency and tumor selectivity of *C*. *novyi*-NT, coupled with the development of localized drug delivery systems such as liposomal carriers, hold the potential to further improve the therapeutic window and broaden its applicability in the treatment of solid malignancies. However, only a limited number of clinical trials have been conducted to evaluate the safety profile and therapeutic efficacy of *C*. *novyi*-NT (Table 4). Potential concerns regarding off-target toxicity, particularly due to uncontrolled bacterial proliferation or systemic dissemination following intravenous or intratumoral injections, remain significant challenges to clinical translation. These safety concerns highlight the critical need for strategies that enable precise tumor targeting and localized colonization of *C*. *novyi* spores. Approaches such as image-guided delivery, biomaterial-based encapsulation, or tumor-specific promoters may enhance spatial specificity, reduce systemic exposure, and improve therapeutic outcomes. Further investigation into pharmacokinetics, tumor selectivity, and host immune responses to *C*. *novyi* is essential to support its advancement into broader clinical applications.

### 3.3. Combination Therapy: IRE Ablation and C. novyi-NT Bacterial Therapy

The combination of IRE ablation and *C*. *novyi*-NT bacterial therapy presents a promising strategy for treating CRLM by leveraging their complementary mechanisms of action. IRE induces transient hypoxia within the TME, which may facilitate the germination and cytotoxic activity of the obligatory anaerobe *C*. *novyi*-NT. In addition, both modalities possess the capacity to elicit anti-tumor immune responses [9,10].

Transarterial delivery of *C*. *novyi*-NT was explored as a method to potentially minimize systemic toxicity [73]. Roberts et al. demonstrated that intratumoral injection of *C*. *novyi*-NT spores induces a precise, localized lytic effect on hypoxic tumor regions in preclinical models, including a rat orthotopic brain tumor model and spontaneous solid tumors in companion dogs. In the canine study, the treatment was well tolerated and yielded objective responses in 37.5% of cases, with complete responses in three dogs. Translating this approach to a human patient with advanced leiomyosarcoma resulted in marked tumor regression, suggesting therapeutic potential. While acquired immune responses were not clearly demonstrated, the pronounced innate immune activation observed supports further clinical investigation of *C*. *novyi*-NT as a tumor-selective, bacteriolytic agent capable of eliciting immunogenic antitumor responses [73].

To enhance the precision and efficacy of *C*. *novyi*-NT therapy for CRLM, a promising approach involves transcatheter intrahepatic artery infusion, which offers advantages over conventional intravenous or intratumoral delivery. Preclinical studies have explored the use of clinical-grade *C*. *novyi*-NT spores, prepared under stringent regulatory guidelines for sterility and viability [79], delivered via intrahepatic artery with embolization in relevant animal models. Notably, in rat liver tumor models, intrahepatic arterial delivery with embolization demonstrated significantly superior tumor growth inhibition compared to direct intratumoral injection at early time points post-treatment [80]. Histological evaluation, utilizing standard staining techniques, confirmed robust spore germination specifically within the hypoxic regions of the tumors, accompanied by significant liquefactive necrosis and minimal evidence of systemic bacterial dissemination, indicating a high degree of tumor-specific delivery. Consistent with these findings, studies in other liver tumor models have similarly shown that intrahepatic arterial delivery of *C*. *novyi*-NT results in targeted spore localization and potent oncolytic activity, with a favorable safety profile characterized by a lack of significant off-target effects in normal liver tissue [80]. These collective preclinical findings underscore the superior precision and therapeutic efficacy of intrahepatic arterial delivery for *C*. *novyi*-NT, providing a strong rationale for its integration with other locoregional therapies, such as IRE ablation, to potentially enhance treatment outcomes for CRLM.

When combined with IRE, *C*. *novyi*-NT therapy may yield multiple synergistic benefits. First, IRE-induced hypoxic zones create ideal niches for selective germination and proliferation of *C*. *novyi*-NT. Second, the release of tumor-associated antigens (TAAs) by IRE can be amplified by the immunostimulatory effects of *C*. *novyi*-NT, enhancing local and systemic anti-tumor immunity [81]. Third, this dual approach may help control distant metastases, supported by preclinical evidence showing synergy between ablation therapies and immuno- or bacteriolytic agents [72,82,83].

**Figure 1:**
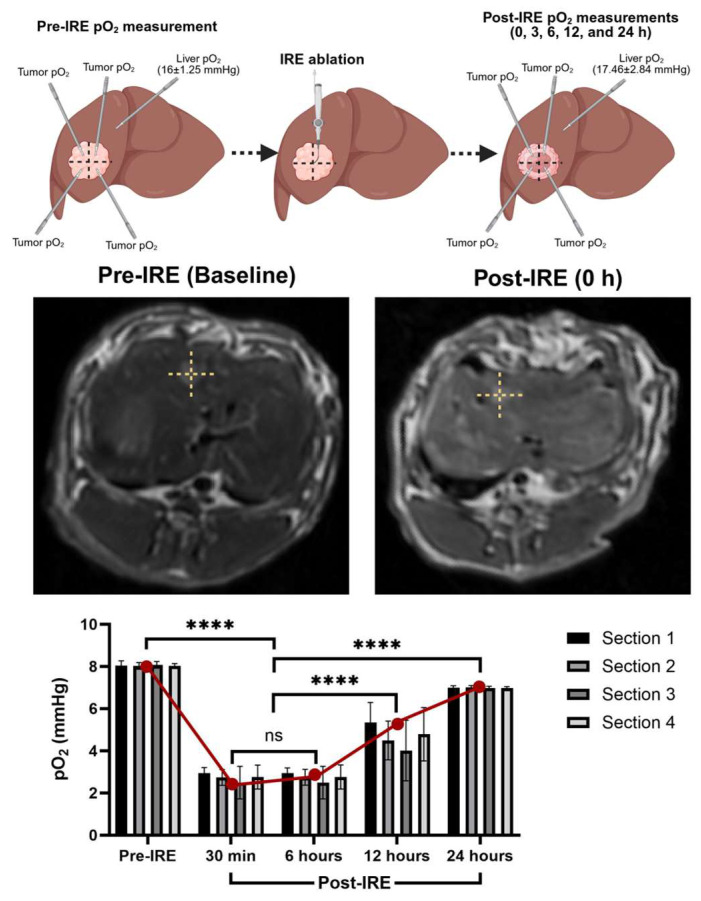
Tumor pO_2_ measurements significantly change following IRE ablation, lasting for a couple of hours. After post-IRE 24 h ablated tumor may exhibit moderate pO_2_ rebound and early tissue remodeling. (**** *p* < 0.001).

Intrahepatic administration of *C*. *novyi*-NT spores with embolization may confine the spores within the tumor, prolong hypoxia, and minimize systemic spread. Notably, studies have reported no detectable spores in the bloodstream beyond 48 h post-treatment. In a previous study, we observed that oxygenation measurement systems and hypoxia markers consistently show significant reductions in tumor partial pressure of oxygen (pO_2_) within 30 min to 6 h following IRE ablation (Figure 1). Importantly, normal liver tissue oxygenation remained largely unaffected by IRE, suggesting a degree of tumor-specific hypoxic induction. This transient hypoxic TME, strategically timed, is hypothesized to enhance *C*. *novyi*-NT germination, particularly when spores are delivered via the hepatic artery.

The synergistic integration of IRE ablation and intrahepatic arterial delivery of *C*. *novyi*-NT spores with embolization represents a significant advancement in the delivery of this engineered anaerobic bacterium. *C*. *novyi*-NT selectively colonizes hypoxic tumor regions, inducing localized tumor lysis and systemic immune activation while sparing healthy, oxygenated tissues [10]. In contrast, traditional delivery methods such as intravenous or intratumoral injection are limited by rapid immune clearance and systemic toxicity. Administering *C*. *novyi*-NT following IRE-induced apoptosis and hypoxia may allow the spores to be retained within the altered TME, target hypoxic regions more effectively, and avoid premature clearance by the host immune system. Furthermore, IRE disrupts tumor microvasculature, augmenting hypoxia and enhancing immune infiltration, thus creating a favorable environment for *C*. *novyi*-NT germination [84].

Multiparametric MRI, PET-CT, and contrast-enhanced ultrasound assess tumor characteristics [85]. MRI sequences, including T1/T2-weighted (T1w/T2w) imaging, diffusion-weighted imaging (DWI), and dynamic contrast-enhanced (DCE) MRI, play a crucial role in guiding interventional procedures [86]. In a previous preclinical study, Hu et al. demonstrated the immediate effects of IRE on tumor microstructure and perfusion, which are detectable within hours using T1w, T2w, DWI, and DCE-MRI in a rodent liver tumor model [86]. The increase in the apparent diffusion coefficient parameter derived from DWI reflected changes in cell membrane permeability and apoptosis, while DCE-MRI revealed reduced perfusion in the ablated zones, findings that were validated by necrosis and immune cell infiltration on histological examination [87]. Furthermore, PET/MRI has been utilized to identify metabolic shifts that are predictive of treatment outcomes, establishing multiparametric MRI as a non-invasive tool for assessing the effects of IRE [88]. In a previous study, Eresen et al. investigated the potential value of multiparametric MRI for distinguishing temporary from permanent tissue changes following IRE, a critical step for early evaluation of treatment efficacy [89]. Utilizing advanced statistical learning models and quantitative data from conventional T1w and T2w MRI scans, the study accurately identified IRE ablation regions in a preclinical rabbit model. The developed Support Vector Machine (SVM) classifiers demonstrated particularly high accuracy, with detection rates of 91.06% for T1w, 96.15% for T2w, and 98.04% for combined MRI data, underscoring their potential for immediate assessment of therapeutic outcomes. Furthermore, Cillis et al. investigated the high-frequency IRE to optimize electrode characteristics and predict the ablated area, thereby enhancing therapeutic procedure efficiency and reducing treatment time [90]. These MRI techniques hold significant potential for real-time monitoring of IRE-induced hypoxia and subsequent immune activation in the context of combined IRE and *C*. *novyi*-NT therapy.

**Figure 2:**
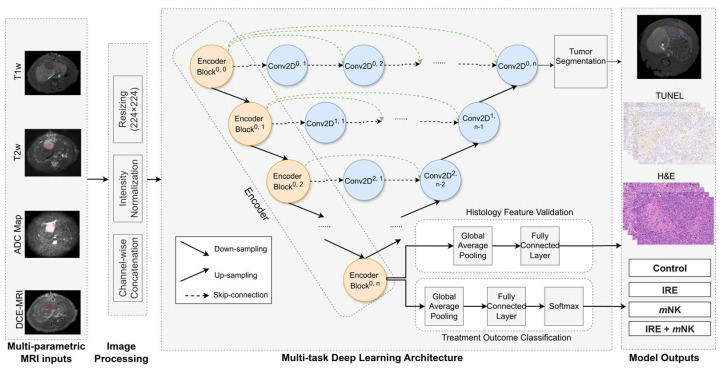
Architecture of the multi-task deep learning model. The U-Net++ model incorporates an EfficientNet-B0 encoder to extract hierarchical imaging features from MRI slices (orange blocks). The decoder ( blue blocks) restores spatial resolution using nested skip connections (dashed arrows) to generate tumor segmentation masks. Separate classification processes encoded features to predict treatment outcomes. Arrows indicate the direction of data flow.

Quantitative analysis of the medical imaging approaches have shown promise in predicting survival in CRLM [91], while AI models have demonstrated high accuracy (>90%) in detecting metastatic lesions [92]. In the context of the proposed combination therapy, AI could potentially optimize the timing of *C*. *novyi*-NT delivery based on the evolving TME post-IRE ablation. Recently, our group developed a multi-task deep learning model designed to analyze 3D multiparametric MRI data and identify image biomarkers that reflect TME changes in response to immunotherapies (Figure 2). This model employs encoder–decoder architecture, where the encoder predicts treatment response and TME alterations (validated against histology), while the decoder simultaneously performs tumor segmentation. This comprehensive approach may offer the potential for early, non-invasive assessment of treatment effects, facilitating timely therapy adjustments and ultimately improving outcome prediction, utilizing standard MRI protocols and thus offering a cost-effective and clinically translatable solution without the need for novel imaging technologies.

**Figure 3:**
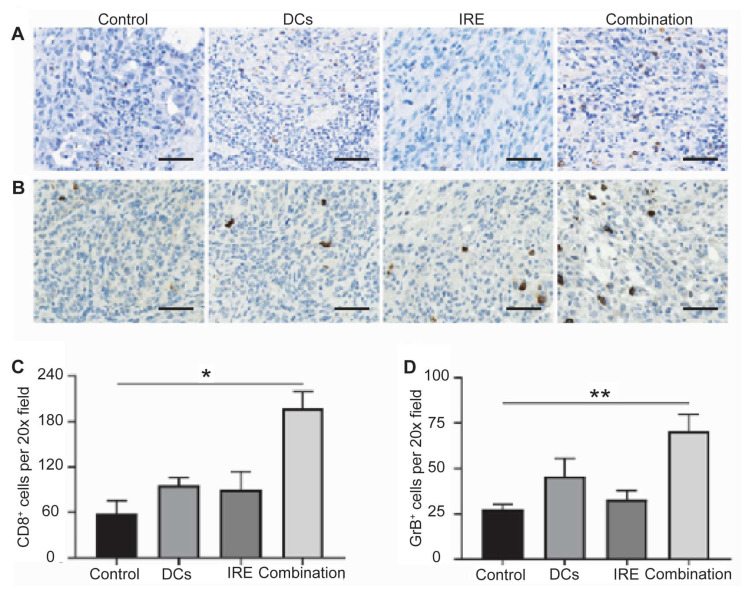
Intratumor immune responses of the IRE ablation and dendritic cell vaccination combination. Pancreatic ductal adenocarcinoma tumor CD8 (**A**) and granzyme B (GrB) (**B**) staining after different treatments. Relative quantification of CD8^+^ (**C**) and GrB^+^ (**D**) cells in pancreatic tumors for each group. Scale bars represent 40 μm. (* *p* < 0.05, ** *p* < 0.01) (Adapted from Yang et al. [93]).

The immunomodulatory effects of IRE ablation have been characterized in preclinical models [93,94]. IRE induces immunogenic cell death, leading to the release of TAAs and damage-associated molecular patterns (DAMPs), which promote the recruitment and activation of immune effector cells. In rodent models of hepatic tumors, IRE consistently stimulated a rapid infiltration of natural killer cells, CD8^+^ cytotoxic T lymphocytes, CD11c^+^ dendritic cells, and F4/80^+^ monocytes within 24 h, exceeding the immune cell infiltration observed after cryoablation [95]. These findings were further corroborated by immunohistochemistry and flow cytometry, which showed increased infiltration of CD8^+^ and granzyme B^+^ cytotoxic cells, indicating both local and systemic immune activation (Figure 3). These immune responses suggest that IRE enhances immune cell trafficking into the TME and reduces immunosuppressive components [89,93,96,97,98,99,100,101].

Moreover, studies combining IRE with immunotherapies, such as dendritic cell vaccines, have demonstrated additive effects [93,97,101], further supporting the rationale for combining IRE ablation with *C*. *novyi*-NT therapy for CRLM.

By applying advanced machine learning algorithms to multiparametric MRI data, these models can quantify critical features such as hypoxia, microvascular disruption, and immune cell infiltration following IRE and *C*. *novyi*-NT therapy. Unlike conventional imaging, AI-enhanced biomarkers can detect early perfusion and structural changes, enabling real-time assessment of therapeutic response. This approach may significantly improve clinical decision-making by identifying responders early, optimizing treatment protocols, and reducing reliance on invasive biopsies. Ultimately, this framework supports the development of scalable, personalized monitoring strategies that advance precision oncology in the context of bacteriolytic and ablative therapies.

## 4. Conclusions

The integration of IRE ablation with *C*. *novyi*-NT bacterial therapy represents a transformative approach for CRLM, addressing the critical challenge of hypoxic tumor resistance. IRE ablation disrupts tumor microvasculature, creating a hypoxic niche ideal for *C*. *novyi*-NT germination, while both modalities stimulate robust local and systemic immune responses. Preclinical studies demonstrate that IRE induces immunogenic cell death, with significant infiltration of CD8^+^ CTL cells, natural killer cells, and macrophages within 24 h, surpassing cryoablation [95]. The addition of intrahepatic arterial delivery with embolization enhances *C*. *novyi*-NT specificity, achieving superior tumor growth inhibition compared to intratumoral administration, with minimal systemic toxicity. This synergy holds the potential to downstage unresectable CRLM, potentially integrating with portal vein embolization or associating liver partition and portal vein ligation to improve resectability potential. Advanced imaging, including multiparametric MRI and AI-driven radiomics, further optimizes this approach by enabling real-time monitoring of hypoxia and immune activation, improving treatment precision.

Several challenges remain that necessitate further investigation. Optimizing the timing, dosing, and administration routes of IRE ablation plus *C. novyi*-NT bacterial therapy is crucial to maximizing efficacy and safety. The inherent risk of complications, such as arrhythmias associated with IRE ablation or potential systemic toxicity from *C*. *novyi*-NT, underscores the need for rigorous preclinical validation. Clinical translation will require comprehensive phase I/II trials to establish robust safety profiles and demonstrate clear therapeutic benefits in patients, particularly those with currently unresectable CRLM. Compared to existing therapeutic modalities such as systemic chemotherapy or thermal ablation, this combination offers the potential for reduced systemic toxicity and enhanced systemic control through immune activation, thereby addressing key limitations associated with hypoxic resistance and high recurrence rates. The integration of AI-derived MRI biomarkers promises to further enhance clinical decision-making by providing non-invasive, real-time prediction of treatment responses, potentially reducing the reliance on invasive biopsies for monitoring. Overall, this innovative strategy holds significant promise for revolutionizing the management of CRLM by strategically leveraging TME manipulation and immune priming, but its ultimate success hinges on overcoming translational hurdles through comprehensive preclinical and clinical validation.

Future management of CRLM will likely prioritize personalized, multimodal therapeutic strategies. The utilization of genomic and radiomic biomarkers could play a critical role in guiding patient selection for IRE and *C*. *novyi*-NT therapy, thereby optimizing individual treatment outcomes. Combining these regional and bacteriolytic approaches with systemic immunotherapies, such as immune checkpoint inhibitors, may further amplify systemic anti-tumor immunity and improve the control of distant metastases. The development and application of AI-driven models, integrating multiparametric MRI and histological data, will be essential for enabling real-time therapy adjustments and enhancing treatment precision. Furthermore, the development of scalable AI biomarkers could potentially reduce costs and improve global access to advanced diagnostic and monitoring tools. Future clinical trials must rigorously validate the efficacy and safety of these integrated approaches while also proactively addressing issues of equitable access to ensure widespread adoption across diverse healthcare settings.

CRLM presents a formidable challenge in oncology, with current treatment strategies often constrained by limited resectability and the development of resistance to conventional therapies. The novel combination of IRE and *C*. *novyi*-NT bacterial therapy, enhanced by advanced imaging modalities and AI-driven analytics, offers a promising solution to overcome these limitations. By strategically exploiting the unique ability of IRE ablation to induce tumor hypoxia and stimulate anti-tumor immune responses, paired with *C*. *novyi*-NT’s targeted lytic activity within hypoxic tumor regions, this integrated approach directly targets the critical barriers in the effective management of CRLM. Furthermore, established surgical techniques, including portal vein embolization and associating liver partition and portal vein ligation, can further support the potential for tumor resectability in conjunction with this novel therapy. The integration of AI-driven analytics into advanced imaging holds the key to optimizing treatment precision through real-time monitoring of hypoxia and immune activation. However, rigorous preclinical and subsequent clinical studies are essential to comprehensively confirm the safety profile, therapeutic efficacy, and long-term outcomes associated with this innovative combination. If successfully validated in human trials, this integrated strategy has the potential to fundamentally redefine the treatment paradigm for CRLM, ultimately leading to improved survival rates and enhanced quality of life for patients afflicted with this devastating disease.

## Figures and Tables

**Table 1:** Advantages and limitations of the currently utilized treatment strategies for CRLM.

Treatment	Advantages	Limitations
Surgical Resection	High 5-year survival (30–58%)	Limited eligibility (10–20%)
Systemic Chemotherapy	Extends survival (20–24 months)	Hypoxic resistance, toxicity
Radiofrequency/Microwave ablation	Effective for small tumors	Size/location constraints
Transarterial chemoembolization	Downstages tumors	Limited survival benefit
Stereotactic Body Radiation Therapy	High local control	Uncertain survival benefits

**Table 2:** Mechanistic evaluation and limitations of the IRE ablation.

Aspect	Details	Limitations
Mechanism	Non-thermal ablation	Limited to small tumors
Efficacy	~70% local control at 1 year	Long-term data limited
Safety	Low morbidity/mortality	Requires general anesthesia
Applications	Tumors near critical structures	Technical complexity

**Table 3:** Mechanism of *C*. *Novyi*-NT bacterial therapy and challenges.

Aspect	Details	Limitations
Mechanism	Targets hypoxic regions	Delivery optimization needed
Efficacy	Tumor lysis, immune activation	Limited human data
Safety	Well-tolerated in early trials	Potential systemic toxicity
Applications	Hypoxic tumors	Combination strategies untested

**Table 4:** The list of clinical trials registered to the United States National Library of Medicine (www.clinicaltrials.gov) for the treatment of solid tumors using *C*. *novyi*-NT.

Study Title	Phase	Administration	Treatment	Status	Identifier
Pembrolizumab with intratumoral injection of *Clostridium novyi*-NT	Phase 1	Intratumoral	*C*. *novyi*-NT Pembrolizumab Doxycycline	Active, not recruiting	NCT03435952
Safety study of *Clostridium novyi*-NT spores to treat patients with solid tumors that have not responded to standard therapies	Phase 1	Intravenous	*C*. n*ovyi*-NT	Terminated	NCT01118819
Safety study of intratumoral injection of *Clostridium novyi*-NT spores to treat patients with solid tumors that have not responded to standard therapies	Phase 1	Intratumoral	*C*. *novyi*-NT	Completed	NCT01924689
One time injection of bacteria to treat solid tumors that have not responded to standard therapy	Phase 1	Intravenous	*C*. n*ovyi*-NT	Terminated	NCT00358397

## Abbreviations

The following abbreviations are used in this manuscript:

CRLM	Colorectal cancer liver metastasis
*C. novyi*-NT	*Clostridium novyi*-NT
DCE	Dynamic contrast-enhanced
DWI	Diffusion-weighted imaging
EGFR	Epidermal growth factor receptor
IRE	Irreversible electroporation
TME	Tumor microenvironment
